# Temporal overlap and repeatability of feather corticosterone levels: practical considerations for use as a biomarker

**DOI:** 10.1093/conphys/cow051

**Published:** 2016-11-09

**Authors:** Christopher M. Harris, Christine L. Madliger, Oliver P. Love

**Affiliations:** Department of Biological Sciences and the Great Lakes Institute for Environmental Research (GLIER), University of Windsor, Windsor, Ontario, Canada N9B 3P4

**Keywords:** Biomarker, corticosterone, feather, glucocorticoid, repeatability

## Abstract

The measurement of corticosterone (CORT) levels in feathers has recently become an appealing tool for the conservation toolbox, potentially providing a non-invasive, integrated measure of stress activity throughout the time of feather growth. However, because the mechanism of CORT deposition, storage and stability in feathers is not fully understood, it is unclear how reliable this measure may be, especially when there is an extended interval between growth and feather collection. We compared CORT levels of naturally grown feathers from tree swallows (*Tachycineta bicolor*) that were moulted and regrown concurrently and therefore expected to have similar CORT levels. Specifically, we compared the same feather from the left and right wing (moulted symmetrically) and different types of feathers (wing, back and tail) expected to have been moulted within the same time period. We found that larger, heavier feathers held more CORT per unit length. In addition, we found a lack of concordance in CORT levels both within the same feather type and between different feather types, even after taking into account differences in feather density. Our results indicate that naturally grown feathers may not consistently provide an indication of stress status. Additionally, conflict in results may arise depending on the feather assayed, and total feather volume may be an important consideration when interpreting feather CORT levels. Future work is necessary to determine explicitly the mechanisms of CORT deposition, the effects of environmental exposure and feather wear on the permanence of the feather CORT signal, and the influence of responses to wild stressors on feather CORT levels, before feather CORT can be implemented effectively as a tool for ecological and conservation applications.

## Abstract (precis)

We evaluated whether feather corticosterone represents a straightforward indicator of stress in birds by investigating the consistency of levels extracted from multiple tree swallow feathers grown at the same time but at different body locations. We found that different feathers contained different levels, complicating categorization of low- and high-stress individuals.

## Introduction

The use of physiological measures as biomarkers of environmental change and disturbance in species of conservation importance has been proposed to be a powerful tool for practitioners (Cooke *et al*., 2013). To be effective in this capacity, potential measures need to be consistent and reliable indicators of condition or intrinsic state (Madliger and Love, 2014). Glucocorticoid (GC) activity has been suggested as one such biomarker because of the role of GCs in daily energy balance and in response to acutely stressful events (Landys *et al*., 2006; McEwen and Wingfield, 2010; Dantzer *et al*., 2014). However, measuring GCs in the circulation can be difficult, invasive and limited to certain time periods; issues that are especially undesirable in a metric directed towards species of conservation concern (Sheriff *et al*., 2011). As a result, a number of alternative, less invasive sampling media have been proposed and tested (i.e. faeces, saliva and keratin integuments; Sheriff *et al*., 2011). Of these, hormone extraction from feathers is a promising, but currently less understood method (Bortolotti *et al*., 2008). In particular, determining the mechanisms of corticosterone (CORT) deposition into feathers, the specificity of assays/antibodies, hormone stability over time, mass dependency, influences of feather colour and type, and variation in CORT along feather length are all necessary in order to understand fully how to interpret feather CORT levels within and across individuals (Lattin *et al*., 2011; Jenni-Eiermann *et al*., 2015; Berk *et al*., 2016; Romero and Fairhurst, 2016).

**Figure 1:**
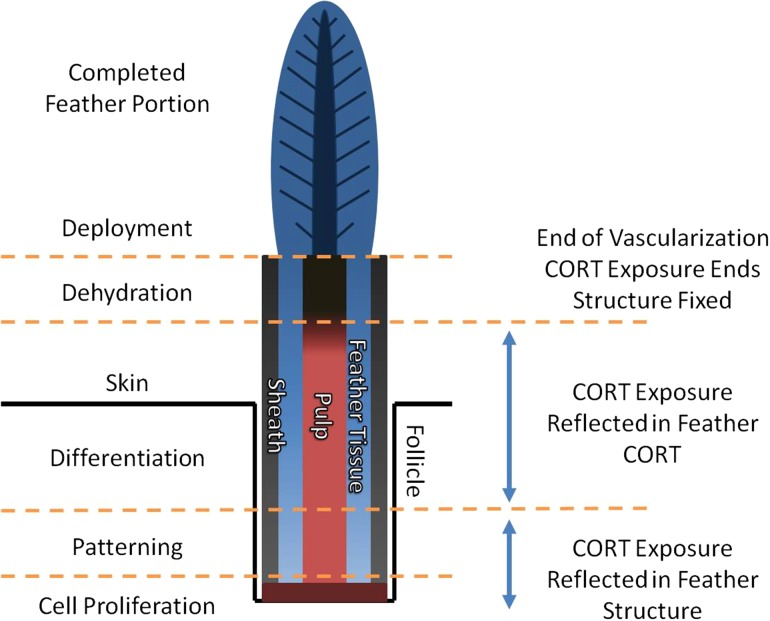
Diagram representing our current understanding of corticosterone deposition into the longitudinal cross-section of a simplified and idealized feather at mid-growth. Cells proliferate at the base of the feather follicle, pushing previously grown feather cells upwards (Maderson *et al*., 2009). The cells pattern and differentiate as they move upward through the follicle, forming an inner vascularized dermal core, surrounding feather tissue and an outer sheath (Stettenheim, 2000). Once beyond the skin, feather tissues are completed, and the dermal core recedes, leaving a pulp cap as its remnant (Lin *et al*., 2006). All tissues dehydrate, and the outer sheath and pulp caps are removed by friction and preening, deploying completed feather tissues (Stettenheim, 1972). Corticosterone (CORT) exposure during early feather growth results in changes to feather structure because of interference with protein production, whereas exposure later in development leads to CORT becoming entrapped within feather tissues and thus reflected in feather CORT levels (Jenni-Eiermann *et al*., 2015). Corticosterone exposure ends with the completion of vascularization (Bortolotti *et al*., 2008).

The currently proposed model of GC deposition in feathers involves entrapment of CORT, the primary avian GC, as it circulates in the vascularized section of the feather pulp that supplies nutrients and other resources to surrounding structures during feather growth (Bortolotti *et al*., 2008; Jenni-Eiermann *et al*., 2015). This process takes place in a growing feather between the area of cell proliferation at the base of the feather follicle and the area of pulp recession preceding feather deployment (Maderson *et al*., 2009; Fig. 1); circulating CORT levels can be deposited throughout this blood quill (Jenni-Eiermann *et al*., 2015). Once pulp caps are formed, this section of the feather is no longer vascularized, and CORT entrapped within the feather is assumed to be held securely until sampling and analysis of the fully grown feather (Bortolotti *et al*., 2009).

The longer time of integration of integument CORT, when compared with other measures such as blood or faeces, should result in this measure being less sensitive to short-term perturbations or concentration changes. This is because the CORT level from a full feather is expected to represent the average circulating level during the entire period of feather growth (i.e. a period of weeks rather than minutes or hours; Bortolotti *et al*., 2009). As moult occurs in a defined sequence at fixed and predictable intervals, with multiple feather tracts regrowing simultaneously during heavy periods of moult, the analysis of feathers grown concurrently and sequentially offers a method of testing the reliability of feather CORT to reflect stress exposure. For example, the weeks-long period of integration over the time of growth leads to inherent insensitivity of the total feather CORT level to any single short-term event, suggesting that feathers which overlap closely in growth time, but are found at different locations on the bird, should also show strong agreement in levels. If this understanding of CORT deposition into feathers is correct, a chronic environmental stressor experienced by a bird should translate into high CORT levels in all feathers grown at the same time. This property is necessary in order for feather CORT to be interpreted as a relevant and robust indicator of past exposure to chronically elevated stress levels.

To date, multiple studies have shown that feather CORT most often relates to measurements of circulating CORT levels after a standardized stressor rather than those measured at baseline circulating levels (Bortolotti *et al*., 2008; Lattin *et al*., 2011; Fairhurst *et al*., 2013; Jenni-Eiermann *et al*., 2015). Although this apparent deposition bias towards stress-induced levels may be attributable to the difference in magnitude between baseline and acute levels (Fairhurst *et al*., 2013), it nevertheless calls into question the interpretation of feather CORT as an average of circulating levels during feather growth. Given that chronic stress can lead to a variety of changes in hypothalamic–pituitary–adrenal functioning and reactivity within and across species (Dickens and Romero, 2013), and the stress axis is likely to be down-regulated during moult (Romero, 2002; Romero *et al*., 2005), the circumstances and/or threshold under which environmental challenge may lead to increased feather CORT levels remains unclear. In addition, the short duration of the acute increase in CORT during the stress response in relationship to the duration of feather growth suggests that feathers which overlap significantly, but not completely, in growth may have very different exposures during stressful events. Therefore, it is currently unclear to what extent feather CORT can be expected to be consistent throughout the naturally grown feathers of an individual in the wild. Consequently, evaluation of the assumed consistency of CORT levels among concurrently grown feathers is important for the correct interpretation of feather CORT results and informed feather sampling decisions.

It should also be noted that while feather CORT should be consistent across feathers, it is not required to have equal levels in absolute terms, as differences in size, shape, colour, structure and growth rate could result in different levels per unit of length (Jenni-Eiermann *et al*., 2015; Patterson *et al*., 2015; Romero and Fairhurst, 2016). Lattin *et al*. (2011) also found that sample mass affects the amount of CORT detected, with smaller than expected amounts of CORT detected as the amount of sample increases. Importantly, this apparent mass dependency of the extraction cannot be overcome by the addition of more solvent, suggesting that hormone levels measured from feathers of very different sizes are not directly comparable owing to differences in extraction efficiency (Berk *et al*., 2016). Even within a given feather, the complexity of feather structure means CORT levels may appear to change along the length of the integument depending on whether hormone levels are adjusted by mass or length (Bortolotti *et al*., 2008, Supplemental Materials; Bortolotti *et al*., 2009), although this understanding is potentially complicated owing to different amounts of keratin along a feather's length interacting with the mass dependency of the extraction. Thus, while CORT is expected to be deposited in a time-dependent manner reflecting growth (Bortolotti 2010), feathers of differing size and/or shape may have different capacities. As a result, when comparing different feathers, feather CORT levels should instead have similar levels in relative rather than absolute terms (i.e. in comparison to conspecifics, an individual with prolonged high circulating CORT levels should have correspondingly high relative feather CORT levels in all feathers grown together during this elevation, although absolute levels in these feathers may not be the same).

Here, we investigate patterns of feather CORT levels across feather groups and assess the symmetry and consistency of CORT levels in wild adult tree swallow (*Tachycineta bicolor*) feathers grown during natural moult. Under the assumption that feather CORT is a consistent and therefore reliable biomarker of stress, we predicted that: (i) different feather types (body, primary, secondary and rectrix) should differ in absolute CORT levels on a per length basis because of differences in size, structure and extraction efficiency; (ii) the same flight feather on both sides of the bird should have the same CORT level because these feathers are moulted symmetrically; and (iii) different types of feathers (i.e. flight and contour) should differ in absolute levels, but should have the same relative levels if they were moulted at the same time (i.e. an individual with high wing feather CORT should also have relatively high body and tail feather CORT if they were moulted at the same time; consistency repeatability should therefore be high).

## Materials and methods

### Feather collection

Feathers were obtained from tree swallows in a system of nestboxes at Ruthven Park National Historic Site (42°58′N, 79°52′W) and Taquanyah Conservation Area (42°59′N, 79°54′W) in Haldimand County, Ontario, Canada. Feathers were collected from 16 adult individuals that died naturally during the 2010–2013 breeding seasons for reasons such as starvation, vehicle collision and conflict with invasive house sparrows (*Passer domesticus*). Birds were found within 24 h of death, and whole feathers were collected if they were not visibly contaminated owing to the manner of death and stored at −80°C until assay. Birds and feathers were collected under Canadian Wildlife Service (Environment Canada) Scientific Permit CA0266. See ‘*Statistical analysis*’ section below for numbers and types of feathers collected for each validation.

### Moult in tree swallows

Tree swallows were selected for this validation because they are a free-living model species (Jones, 2003) that undergoes prebasic moult during migration from July to November and a limited spring moult of chin feathers in some individuals (Stutchbury and Rohwer, 1990). Moult begins immediately subsequent to, or in some cases during, breeding (Hussell, 1983). The species’ wide distribution and resilience to study has led to their extensive use in ecological applications such as impact assessment, where physiological biomarkers would be useful tools (Ghilain and Bélisle, 2008; Harms *et al*., 2010; Custer, 2011; Paquette *et al*., 2013; Cruz-Martinez *et al*., 2015). Finally, tree swallow flight feathers are uniformly dark, preventing confounding effects of pigment differences when comparing feather CORT levels (Jenni-Eiermann *et al*., 2015).

Stutchbury and Rowher (1990) detailed tree swallow moult of all feather tracts in relationship to primary feather moult because the moult begins with the innermost primary (P1) in mid-July and progresses in sequence outward to completion (P9) by November. Inner secondary feather moult begins concurrently with moult of P2–P4, progressing in a sequence of S8, S9, S7, while the remaining secondaries are moulted beginning from the outermost secondary (S1; concurrently with P5–P6) inward. The central rectrices begin to be replaced when P3–P5 are being moulted, and tail moult proceeds outwards. Body moult starts with the back, belly and breast feathers when the outermost primary in active moult is P2–P4 and is normally completed by the time flight feathers are fully grown. Moult normally proceeds in all feather tracts without reversal or interruption through the autumn migration. We used this detailed description of moult timing and sequence to determine the feathers most likely to be growing concurrently in our subsequent analyses (below).

### Feather preparation and hormone assay

To remove surface contaminants before analysis, intact feathers were washed by immersion and swirling in a 50 ml Falcon tube filled with a dilute (1%) soap and ultrapure water solution for 30 s (e.g. Bortolotti *et al*., 2008; Jenni-Eiermann *et al*., 2015). Feathers were then rinsed using ultrapure water to remove all soap solution and allowed to air-dry overnight. The calamus was removed from the feather using a razor blade, the remaining feather length was measured with callipers, and feathers were minced into fine (<1 mm) pieces using scissors. Feather pieces were collected in a weighed glass scintillation vial, and the vial was weighed a second time to determine the mass of the feather available to be extracted. Corticosterone was extracted from the minced feathers according to the protocol outlined by Bortolotti *et al*. (2008) using 10 ml of HPLC grade methanol. Samples were sonicated for 30 min and then placed in a 50°C water bath overnight. Feather pieces were removed from the hormone extract by vacuum filtration, after which the methanol was evaporated in a fume hood. Samples were reconstituted using assay buffer and assayed in triplicate using Enzo Life Sciences Corticosterone Enzyme Immunoassay (ADI-901-097). This kit has been previously validated for the measurement of feather CORT (Bourgeon *et al*., 2014). Assayed samples showed an intra- and interassay coefficient of variation of 4.22 and 13.78%, respectively. Feather CORT levels were expressed per length of feather analysed, because this measure is commonly used and thought to reflect incorporation rates during feather growth (Bortolotti *et al*., 2009).

### Statistical analyses

#### Patterns across feather groups

Given that absolute feather CORT levels may vary as a result of feather size, structure and/or extraction efficiency, we examined feather CORT levels across different feather types. To do so, we first compared mean CORT levels obtained from the following four feather regions: primary and secondary feathers of the wing, rectrix feathers from the tail and body feathers from the back. Feather group means for 12 individuals were calculated using levels from a representative selection of feathers for each feather group to avoid the need to assay every feather on every bird. The mean for primaries is composed of levels from primaries P2, P4, P6 and P8 from the right side of an individual. The mean for secondaries is composed of levels from right secondaries S1, S2, S4 and S8, while the mean for rectrices was composed of levels from right rectrices R1, R3 and R5. Back feathers were extracted and assayed as five pooled feathers owing to their small size; as such, the level obtained from the assay already represents the mean level. Given that all four feather groups are from the same 12 individuals and the data could not be normalized across groups using transformations, groups were compared using a Friedman test blocked for individual identity (Friedman, 1937). This analysis was repeated using the weight per unit of length of the feathers in place of CORT levels to examine differences in density across feather types, and groups were again compared using a Friedman test blocked for individual identity. Analyses were completed using R 3.1.3 (R Development Core Team, 2015), and *post hoc* comparisons were performed using a Wilcoxon–Nemenyi–McDonald–Thompson test (Galili, 2010).

#### Feather corticosterone symmetry

Left- and right-side flight feathers across all three flight feather groups were compared to assess the degree to which feathers moulted symmetrically contain the same amount of CORT. This analysis assumes that left and right feathers of the same type are moulted and regrown at the same time. Limited information is available regarding the exact synchronicity of moult; however, symmetrical feather loss is considered an indicator of primary moult (as opposed to unexpected feather replacement; e.g. Marini and Durães, 2001), and many ageing techniques in songbirds are based on the observation of symmetrical wing gaps and matched feather growth (Pyle, 1987). In addition, symmetrical moult of flight feathers is considered to be highly beneficial to the maintenance of flight and has been linked to survival probability (Freed and Cann, 2012), particularly in species heavily dependent on the maintenance of aerodynamic qualities (Balmford *et al*., 1993). Given that swallows forage solely on the wing and migrate during moult, this is likely to be highly pertinent to our study species. Deviations in symmetry in both growth rate and timing of moult may be observed during times of severe environmental challenge (e.g. food limitation; Swaddle and Witter, 1994; Freed and Cann, 2012), in response to lowered body condition (Swaddle and Witter, 1994) or owing to potential asymmetry in the feather follicles themselves (Møller, 1996). Overall, paired feathers of the left and right wing are most likely to be moulted and regrown as symmetrically as possible and therefore provide a robust means of testing the consistency of feather CORT levels within a bird. To minimize the effect of pseudoreplication, the following six representative feathers were chosen from all flight feathers: primaries P2 and P6, secondaries S2 and S4 and rectrices R1 and R5 from both sides were assayed in eight birds. Corticosterone levels in the feathers were compared using a single linear regression including all 48 feather pairs (i.e. all feather types together). The CORT levels of both left and right feathers were normal without transformation.

#### Consistency of feather corticosterone levels

Finally, to assess the consistency of the information provided by feather CORT, the repeatability of CORT levels in six different feathers, expected to be grown naturally at overlapping times in moult, was evaluated using feathers from 16 birds. Repeatability was assessed according to Lessells and Boag (1987). The feathers were chosen to coincide with a heavy period of moult (i.e. a large degree of temporal overlap across several feather types) to allow for a large number of comparisons. Primaries P4 and P5, secondaries S1 and S8, rectrix R1 and back feathers were used because they are all moulted at similar times (Stutchbury and Rohwer, 1990). As absolute levels of different feathers are not directly comparable owing to differences in extraction efficiency across different masses (Lattin *et al*., 2011), as well as differences in feather size, structure, growth rate and possible CORT-holding capacity, levels were standardized by subtracting the mean CORT level of that feather type (i.e. we calculated consistency repeatability; Nakagawa and Schielzeth, 2010; Dingemanse and Dochtermann, 2013; Biro and Stamps, 2015). This allows for the evaluation of the consistency of the signal across feathers relative to those of conspecifics, as an individual with higher relative circulating CORT levels is also expected to have higher relative feather CORT levels in all feathers grown during that time, although none of these levels are directly comparable to each other. The ranked repeatability of these same feather CORT levels was also assessed to determine the within-individual consistency of feather CORT. All analyses were conducted in JMP 10 (SAS Institute).

## Results

### Patterns across feather groups

**Figure 2:**
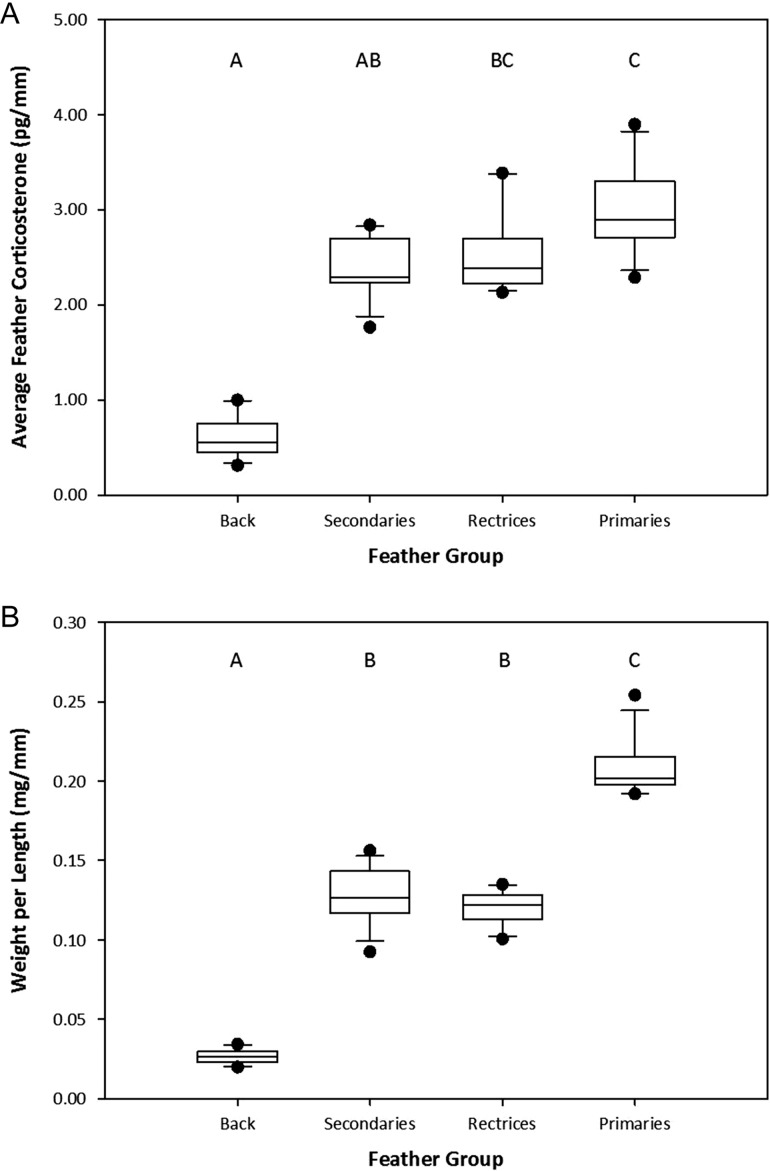
(**A**) Box plot of average corticosterone level (in picograms per millimetre) across different types of feathers in 12 individuals. Letters denote which feather groups are significantly different by the Friedman test (χ^2^(3) = 30.0, *P* < 0.0001) blocked for individual identity. (**B**) Box plot of the average feather weight per unit length (in milligrams per millimetre) for different types of feathers in 12 individuals. Letters denote which feather groups are significantly different by the Friedman test (χ^2^(3) = 32.5, *P* < 0.0001) blocked for individual identity.

The four feather types showed significant differences in feather CORT levels (Friedman test: χ^2^(3) = 30.0, *P* < 0.0001; Fig. 2A), and *post hoc* analysis indicated that, on a per length basis, primary feathers contained more feather CORT than secondary and back feathers, and back feathers contained less feather CORT than rectrices. The mass (in milligrams; mean ± SD) of each feather group was as follows: primaries, 13.84 ± 4.92; secondaries, 5.07 ± 2.00; rectrices, 5.71 ± 1.14; and back, 3.36 ± 0.66. The four feather types in the same samples showed significant differences in weight per unit of length (Friedman test: χ^2^(3) = 32.5, *P* < 0.0001; Fig. 2B), and *post hoc* analysis indicated that given the same length of feather, primary feathers are heavier than secondary feathers and rectrices, and back feathers are lighter than all flight feathers.

### Symmetry and consistency of feather corticosterone levels

**Figure 3:**
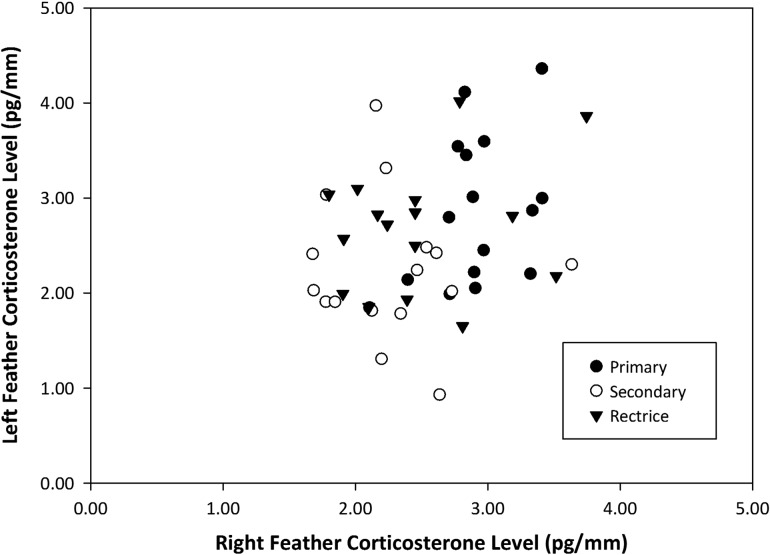
Linear regression of right and left feather corticosterone levels (in picograms per millimetre) in two representative feathers of three feather groups in eight birds (*n* = 48); *P* = 0.05, *R*^2^ = 0.08. Representative feathers were as follows: primaries (P1 and P6), secondaries (S2 and S4) and rectrices (R1 and R5).

We found no relationship between feather CORT levels from the left and right side of a bird (i.e. feathers expected to be grown over identical time frames; *P* = 0.05, *R*^2^ = 0.08; Fig. 3), and the coefficient of determination for the model was low, indicating that feathers moulted at the same time do not have the same feather CORT level. Importantly, our results do not differ if each feather type (primary, secondary and rectrix) are analysed separately.

**Figure 4:**
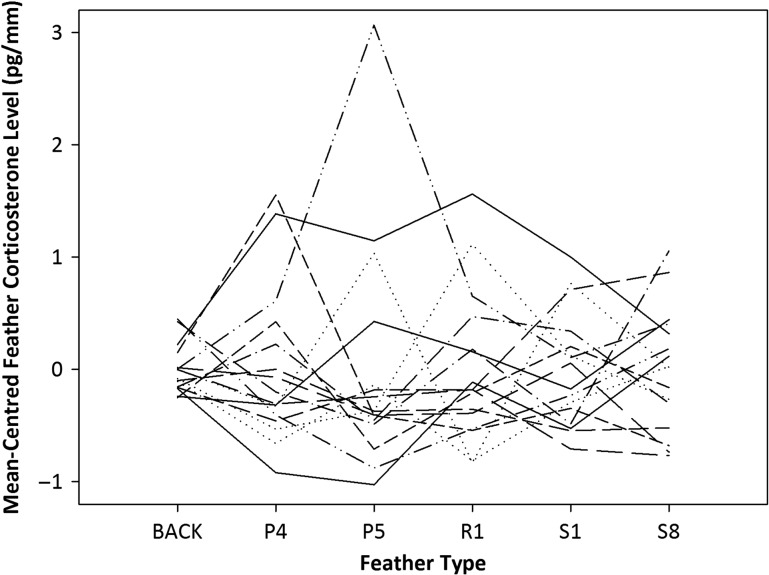
Feather corticosterone levels (in picograms per millimetre) across different feather types grown at a similar time during moult (*r* = 0.24, *F*_15,80_ = 2.87, *P* = 0.001). To improve comparisons between feathers of different sizes, feather corticosterone levels have been mean-centred (see Materials and methods). Each line represents levels from five pooled back feathers, primaries P4 and P5, rectrix R1, secondary S1 and tertial S8 from an individual bird. Under perfect repeatability, individual lines would be horizontal, each with a different intercept.

**Figure 5:**
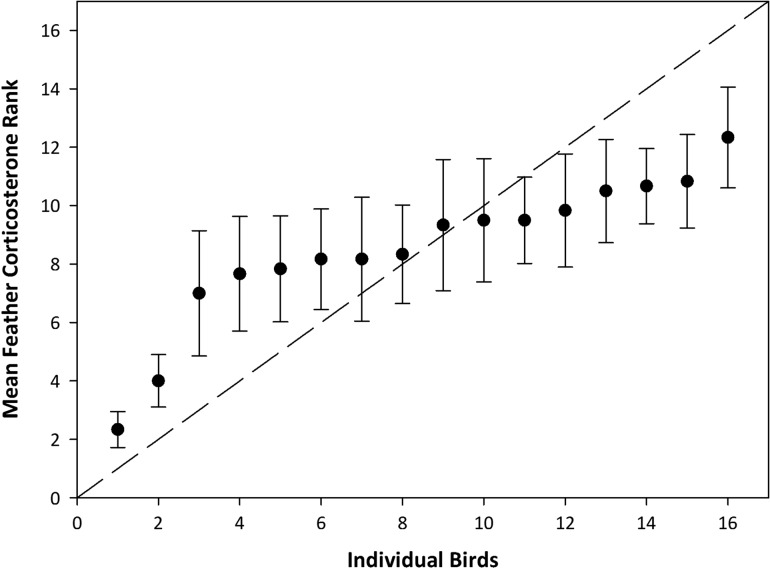
Ranked repeatability of feather corticosterone levels of 16 individuals across six feather types moulted during similar time periods (*r* = 0.15, *F*_15,80_ = 2.08, *P* = 0.02). Points represent mean feather corticosterone rank of the individual using measures from primaries P4 and P5, rectrix R1, secondary S1, tertial S8 and five pooled back feathers. Error bars represent 1 SEM, and the dashed line represents perfect repeatability.

Repeatability of feather CORT levels across various feathers moulted at the same time was low (*r* = 0.24; *F*_15,80_ = 2.87, *P* = 0.001; Fig. 4), as the variation across feathers within individuals was larger than the variation between individuals. *Post hoc* analysis indicated that the lowest six birds were significantly different from the highest bird, and that the two highest birds could be distinguished from the lowest two birds based on feather CORT levels. Overall, 13 birds could not be categorized as either high or low because of within-individual variation. When assessed by rank, the calculated repeatability statistic was found to be lower (*r* = 0.15, *F*_15,80_ = 2.08, *P* = 0.02; Fig. 5).

## Discussion

Feather CORT levels have primarily been used in ecological and conservation contexts as snap shots into aspects of the life cycle that may be hard to observe in wild birds directly. In particular, feather CORT levels are often interpreted as indicators of condition or environmental challenge at the location of moult (e.g. Harms *et al*., 2010; Legagneux *et al*., 2013), predictors of breeding success or survival (e.g. Koren *et al*., 2012) or biomarkers of carry-over effects (e.g. Crossin *et al*., 2013; Bourgeon *et al*., 2014; Harms *et al*., 2015). Feather CORT is particularly appealing for conservation applications because it is minimally invasive, relatively easily collected and stored in field settings, and may be a means to sensitively detect negative (or positive) influences of environmental change on organisms of interest.

During moult, multiple feathers of the same and different types are regrown concurrently in a predictable sequence. As a result, analysing CORT levels of feathers grown simultaneously can provide an indication of whether feather CORT may provide a reliable reflection of exposure to environmental challenge or stress. Likewise, given that feathers regrow over a period of weeks, total feather CORT levels should be inherently insensitive to single, short-term stressors. Therefore, feathers found at different locations on the body but whose growth times overlap should also show agreement in feather CORT levels. Overall, extended environmental challenge should result in high CORT levels across all feathers grown concurrently, and validation of this characteristic is essential to the use of feather CORT as an indicator of prior exposure to chronically elevated CORT levels.

### Patterns across feather groups

In general, we found that larger, heavier feathers held more CORT per unit length, indicating that primary feathers held more CORT than secondary and back feathers, whereas back feathers held less CORT than rectrix feathers. This result is in accordance with our predictions that longer feathers are not only heavier, but heavier per unit length across feather types (also discussed by Bortolotti, 2010), allowing larger feathers to entrap more CORT during the same exposure. Likewise, Patterson *et al*. (2015) found that feather CORT on a per length basis was positively related to feather mass in 10th primaries and primary coverts of Caspian tern (*Hydroprogne caspia*) chicks and suggested that reductions in feather densities attributable to food limitation may reduce feather CORT concentrations. Considering that feathers at opposite extremes of size differed greatly in mass and may have exhibited some mass dependency in their extraction (Lattin *et al*., 2011; Berk *et al*., 2016), it is also possible that the differences between the groups may be larger than those shown here, as any mass dependency experienced would have reduced the levels of the largest feathers relative to the smallest. These results suggest that future studies using feather CORT levels should consider the effect of the total feather volume available for deposition, and that length alone may not always be an adequate proxy for growth in some comparisons. For example, the current model of deposition does not take into account the fact that the total amount of keratin within a feather may vary with feather type and assumes that CORT levels vary stochastically throughout the length of the feather according to variation only in circulating levels (Bortolotti *et al*., 2009). However, this expected pattern requires: (i) the smaller and lighter distal tip of the feather to hold more CORT per unit of keratin than the wider, thicker and heavier feather midsection; (ii) the rachis to hold the same amount of CORT throughout its length regardless of its proximal to distal taper; and (iii) the feather vane to hold the same amount of CORT as the rachis despite its lower volume and mass of keratin. Our results instead suggest that keratin volume should be considered when assessing these patterns and caution the interpretation of comparative levels of sections of a feather when those sections differ markedly in volume and structure.

### Symmetry and consistency of feather corticosterone levels

Matched left and right feathers from six representative feather pairs across all flight feathers did not contain the same feather CORT levels. In addition, calculated repeatability values for six feathers across different regions that overlap in moult timing were low for both relative feather CORT levels (24%) and ranked levels (15%), indicating that there is much larger variation in feather CORT levels within individuals than between (Lessells and Boag, 1987; Boake, 1989). These results suggest that, at least in some species, naturally grown feathers collected long after moult may not reflect the stress status of an individual consistently and that the analysis of multiple feathers may give conflicting results. It is possible that differences in feather CORT among symmetrical feathers may result from differences in growth rate or exact timing of moult (i.e. lack of symmetry). These types of asymmetries are most often associated with food limitation (Swaddle and Witter, 1994; Freed and Cann, 2012). However, researchers would rarely have this information in wild systems and, as our feathers pairs showed no gross differences in size, faults, shape or wear, lack of symmetry is unlikely to explain our results. In addition, we acknowledge that the lack of consistency across different feather types grown at the same time may indicate that the feathers only partly overlapped in growth or differed in growth rate. However, we expected, based on knowledge of moult timing in tree swallows, that these feathers should, at minimum, partly overlap for a period of weeks. If feather CORT is so labile that short time periods can cause marked differences in total feather CORT levels, this poses a difficulty for the use and interpretation of this tool both within and between individuals, particularly for conservation physiology. Depending on which feather is chosen, CORT levels may not adequately reflect the level of environmental challenge being faced by a given individual, and two feathers grown at similar times may provide very different biomarkers of stress level that may not relate to fitness. Although it should theoretically improve consistency, the longer period of GC integration in feathers compared with other media (i.e. plasma, faeces, etc.) does not appear to improve our ability to characterize an individual's stress phenotype.

Our results are similar to those of other studies that have investigated the repeatability of CORT levels of more than one feather from the same individual. For example, CORT levels of different contour feathers from the same individual red-winged blackbirds (*Agelaius phoeniceus*) were not significantly different, but were also not correlated because of high within-individual variation (Kennedy *et al*., 2013). Likewise, on a per mass basis, CORT levels of house finch (*Haemorhous mexicanus*) tail and breast feathers were not significantly different from each other and, although significantly correlated, showed a repeatability of 43% (Lendvai *et al*., 2013). In addition, feather CORT levels were not repeatable within individuals across years in common eiders (*Somateria mollissima*) or snow geese (*Chen caerulescens*; Legagneux *et al*., 2013), and showed a repeatability of 40% in yellow warblers (*Setophaga petechia*) after controlling for a year effect (23% repeatability before controlling for the year effect; Grunst *et al*., 2014).

The relatively low repeatability results for feather CORT are comparable to many of the results found for avian plasma CORT repeatability, which as a whole have been mixed (Romero and Reed, 2008; Wada *et al*., 2008; Ouyang *et al*., 2011; Rensel and Schoech, 2011; Baugh *et al*., 2014). Plasma CORT levels can be assessed over a variety of time frames and tend to be more repeatable over shorter periods (e.g. during breeding compared with across years; Ouyang *et al*., 2011). The repeatability literature on feather CORT has been much more limited, probably owing to the age of the technique and the fact that birds must be caught in subsequent years to assess repeatability (i.e. birds must go through a moult cycle to obtain a second feather CORT sample for repeatability analyses). As a result of this longer time frame, it is perhaps not unexpected to find relatively low repeatability of feather CORT levels, for example, because individuals may experience very different environments during moult on a year-to-year basis. However, further investigation is necessary to determine: (i) whether some individuals may be able to buffer challenges and therefore produce feathers with similar CORT levels despite environmental change; (ii) how differential regulation of the hypothalamic–pituitary–adrenal axis during moult could alter feather CORT deposition within and across species; and (iii) how prior experiences (e.g. breeding effort) may carry over to the moult period to influence feather CORT levels.

### Potential causes of high intra-individual variation

Taken together, these results suggest that different feathers, even when grown at the same time during moult, may not contain as similar levels of CORT as predicted by the current model of deposition. Therefore, feather CORT is either not always a straightforward record of circulating CORT levels during feather growth or the levels are not fixed throughout the life of the feather. As discussed earlier, some of the within-individual variation in feather CORT levels may be the result of an overrepresentation of stress-induced levels experienced during feather growth (Bortolotti *et al*., 2009; Fairhurst *et al*., 2013). As stress-induced GC levels are much higher than baseline levels and are relatively short lived compared with feather replacement duration, feathers with slight differences in growth period may have very different CORT exposure profiles. However, this scenario does not explain the observed lack of correlation in left and right paired feathers. As above, we acknowledge that there are potential differences in the exact period of growth between left and right feathers that could not be accounted for because we were unable to observe moult directly. Future study confirming differences in growth period would be beneficial to our understanding of the potential of feather CORT to differ over short time periods; if two feathers grown over time periods differing by only a few days can show markedly different signals of stress, this biomarker may be hard to interpret in the context of broad environmental quality during moult. Likewise, as the feathers used in this study were grown naturally in adult tree swallows, differences in moult order, timing and growth rate are likely to increase variation. Similar results have been found in the case of stable isotope values in feathers, where such differences can lead to the higher within-individual differences in adult birds when compared with the synchronous moult of nestlings (Carravieri *et al*., 2014). Moreover, this added variation is in addition to the lack of correlation between left and right paired feathers, and the same sources of variation should be expected in many studies of feather CORT in wild birds that undergo moult during inaccessible times. Overall, this high within-individual variation therefore represents a potential barrier to the use of CORT levels in naturally grown feathers as a biomarker of stress in adult birds.

As high circulating levels of CORT are harmful to protein formation, elevated CORT levels during feather growth can have profound negative effects on feather structure that can be maintained throughout the remainder of integument growth (Romero *et al*., 2005; Peters *et al*., 2011; Jenni-Eiermann *et al*., 2015). Given that feathers are necessary for thermoregulation and flight, it follows that birds must minimize CORT-based reductions in feather quality (Jovani and Blas, 2004; Romero *et al*., 2005). Indeed, the seasonal down-regulation of CORT release during moult may be a mechanism to avoid the negative effect of CORT on protein stability and synthesis (Romero *et al*., 2005) and may lead to lower feather CORT levels in general in naturally moulted feathers compared with replaced feathers. However, it is not clear how differences among individuals in their ability to down-regulate hypothalamic–pituitary–adrenal activity or how the current gaps in our understanding of the exact deposition of CORT into feathers (e.g. plasma levels of CORT are in the nanogram range, whereas levels in feathers are in the picogram range; Romero and Fairhurst, 2016) may complicate the interpretation of CORT in feathers grown during natural moult. For example, Done *et al*. (2011) found that reproductive effort (number of young hatched) positively predicted stress-induced CORT levels during moult. It is therefore possible that some of the variation observed in feather CORT levels could be attributable to experiences during breeding carrying over to the subsequent moult stage to influence the regulation of baseline or stress-induced CORT, rather than feather CORT reflecting conditions only during moult.

Despite down-regulation of CORT during moult (Romero, 2002) and the importance of growing high-quality feathers, fault bars (small visible lines caused by structural errors from abnormal feather growth) occur with some frequency (Jovani and Diaz-Real, 2012). Furthermore, it has been suggested that individuals should differentially allocate stress-induced fault bars across feathers to minimize their impacts (Jovani and Blas, 2004). Given that both flight and contour feather tracts are moulted at the same time in many species, differential allocation cannot be accomplished solely through modification of the level of down-regulation, suggesting that there may be further mechanisms to prevent CORT from affecting feather growth in key areas. For example, the mechanisms currently hypothesized to control differential allocation of fault bars, such as blood pressure changes at the follicle collar or constriction of musculature around the follicle (Jovani and Rohwer, 2016), may also be relevant to feather CORT deposition. Additionally, as above, stress-induced changes in feather density may change the ability of the feather to reflect CORT levels (Patterson *et al*., 2015), which may account for the low repeatability of feather CORT.

A second possibility is that initial feather CORT concentrations following moult may be repeatable, but that levels did not remain static between moult and the point at which feathers were collected. It has been shown that GCs in hair can be reduced by washing and weathering following their deposition (D'Anna-Hernandez *et al*., 2011; Hamel *et al*., 2011), and there is evidence that preparatory washes before assay can reduce feather CORT levels (Bortolotti *et al*., 2008; Jenni-Eiermann *et al*., 2015). Indeed, external changes in feather CORT concentrations have been proposed as explanations of discordant results in other studies (Lattin *et al*., 2011; Jenni-Eiermann *et al*., 2015). External changes could result in a lack of repeatability and in the lack of agreement between left and right feathers, because different feathers could have different levels of exposure both within and between feather types owing to placement, function, structure and preening behaviour. In this study, we washed all feathers identically but, if washing itself alters levels stored within the feather, washing treatments may alter our interpretation of repeatability and feather CORT among individuals in general. However, further validation is still necessary regarding the exact deposition mechanisms, differential allocation and stability in order to understand these implications fully.

### Conclusions: feather corticosterone in the context of ecology and conservation

Feather CORT levels in this study were found to differ across feather types as a result of differences in feather density. Left and right paired, symmetrically moulted feathers did not contain the same CORT levels, and the repeatability of CORT levels in different feathers that overlapped temporally during moult was low. Our combined results caution against the use of naturally grown feathers as a reliable indicator of circulating CORT phenotype. We urge further research aimed at determining whether single feathers can be interpreted as whole-organism indicators of stress level or disturbance. It is possible that researchers may need to sample multiple feathers from each individual, only measure feathers of specific types, and that specific knowledge of moult timing and order may be integral to interpreting feather CORT levels accurately and inferring a CORT phenotype. Indeed, detailed knowledge of moult timing may allow researchers to exploit this tool to their benefit, especially in species with a long period of moult, where different feathers could provide information on different aspects of the life cycle.

As conservation physiology aims to contribute to decision-making and policy to foster success, the tools used by the discipline must be well validated to be viable options for practitioners. Our aim here was therefore to raise awareness of an understudied aspect of feather CORT (i.e. repeatability) and to urge researchers and practitioners to consider this validation as they continue to develop the tool. Overall, future work is needed to examine the mechanisms of deposition, external effects, permanence of signal and responses to known stressors in the wild before feather CORT can be used effectively as a tool for conservation and ecological applications.
